# Thumb force deficit after lower median nerve block

**DOI:** 10.1186/1743-0003-1-3

**Published:** 2004-10-19

**Authors:** Zong-Ming Li, Daniel A Harkness, Robert J Goitz

**Affiliations:** 1 Hand Research Laboratory, Departments of Orthopaedic Surgery and Bioengineering, University of Pittsburgh, PA 15213 USA

**Keywords:** Thumb, Hand, Force, Median nerve block

## Abstract

**Purpose:**

The purpose of this study was to characterize thumb motor dysfunction resulting from simulated lower median nerve lesions at the wrist.

**Methods:**

Bupivacaine hydrochloride was injected into the carpal tunnel of six healthy subjects to locally anesthetize the median nerve. Motor function was subsequently evaluated by measuring maximal force production in all directions within the transverse plane perpendicular to the longitudinal axis of the thumb. Force envelopes were constructed using these measured multidirectional forces.

**Results:**

Blockage of the median nerve resulted in decreased force magnitudes and thus smaller force envelopes. The average force decrease around the force envelope was 27.9%. A maximum decrease of 42.4% occurred in a direction combining abduction and slight flexion, while a minimum decrease of 10.5% occurred in a direction combining adduction and slight flexion. Relative decreases in adduction, extension, abduction, and flexion were 17.3%, 21.2%, 41.2% and 33.5%, respectively. Areas enclosed by pre- and post-block force envelopes were 20628 ± 7747 N.N, and 10700 ± 4474 N.N, respectively, representing an average decrease of 48.1%. Relative decreases in the adduction, extension, abduction, and flexion quadrant areas were 31.5%, 42.3%, 60.9%, and 52.3%, respectively.

**Conclusion:**

Lower median nerve lesion, simulated by a nerve block at the wrist, compromise normal motor function of the thumb. A median nerve block results in force deficits in all directions, with the most severe impairment in abduction and flexion. From our results, such a means of motor function assessment can potentially be applied to functionally evaluate peripheral neuropathies.

## Introduction

The thumb has unique anatomical and biomechanical characteristics that are required to perform many manipulative tasks. Thumb motor dysfunction resulting from neuromuscular and musculoskeletal pathologies severely hinders the performance of these daily tasks. Clinical treatment, prevention protocols, and rehabilitation efficacy requires a thorough understanding of thumb motor capabilities, as well as its associated functional deficit. Investigations of underlying pathological mechanism of the thumb help advance clinical treatments such as tendon transfers [1], functional electrical stimulation [2] and plasticity suppression [3].

Measurement of strength during maximum voluntary contraction is a simple and direct means of assessing neuromuscular function. Popular instruments used for quantitative assessment of thumb strength are pinch dynamometers. The pinch output, however, provides limited information about thumb motor function in that it offers a single generic force in one specific direction. Each muscle/tendon within the thumb has a distinct anatomical origin and insertion, suggesting its external force potential in a particular direction [4-6]. Hence, evaluation of strengths in multiple directions offers insight concerning the motor capacity of individual muscles. Force production of a digit has been measured in various directions such as flexion/extension [7,8], abduction/adduction [9-14], or in combined directions [15,16]. Bourbonnais et al. developed an apparatus to measure thumb force production in eight directions in the transverse plane of the thumb and investigated force dependence on the direction of effort [15]. Yokogawa and Hara measured index fingertip forces in various directions within the flexion/extension plane [8]. Recently, we developed experimental apparatuses to measure multi-directional forces of a digit in its transverse plane [17-19]. From these multi-directional forces we constructed force envelopes representative of the characteristic force output pattern of a digit [17-19].

Disorders resulting from traumatic injuries to and various diseases of these nerves are common in clinical practice. Clinical manifestations of hand dysfunction are distinctive depending on the nerve involved. For example, thenar atrophy is a major clinical observation affecting thumb function at the later stages of compression neuropathy of the median nerve. Several studies have been conducted to investigate the effects of simulated peripheral neuropathies using local anesthetization [5,16,20,21]. Kozin et al. [21] studied the effects of median and ulnar nerve blocks on grip and pinch strength and showed significant decreases following nerve blockage [21]. Boatright and Kiebzak [20] investigated the effects of median nerve block on thumb abduction strength. Kaufman et al. [5] measured isometric thumb forces in eight directions together with electromyographic signals of thumb muscles after block of the median nerve. Labosky and Waggy [22] studied the strength related to grip, pinch, thumb adduction, thumb abduction, and finger flexion after radial nerve block [22]. Kuxhaus studied the three dimensional feasible force set at the thumb-tip before and after ulnar nerve block and reported this to be a reproducible and sensitive means to detect impairment.

The purpose of this study was to utilize our developed apparatus and protocols to investigate the effects of lower median nerve lesion on thumb motor function. The lesion was simulated by blocking the median nerve at the wrist using an anesthetic. We hypothesized that a median nerve block would cause (1) a decrease in force production, which would be direction-dependent with the most severe reduction in the abduction direction, and (2) a decrease in the force envelope area and force quadrant area, with the greatest decrease in the abduction quadrant.

## Methods

### Subjects

Six healthy male subjects (mean age: 26.9 ± 5.1 years) participated in this study. The subjects had no previous history of neuromuscular or musculoskeletal disorders of the upper extremities. Each subject signed an informed consent form approved by the Institutional Review Board prior to participating in the experiment.

### Median nerve block

Injections were performed under aseptic conditions while the subjects sat with the forearm supinated and the wrist slightly extended. After the skin at the palmer area of the wrist was cleaned with alcohol, 4 mL of 0.5% bupivacaine hydrochloride (Astra Pharmaceuticals, Westborough, MA, USA) was injected into the carpal tunnel with a sterile 25-gauge short-bevel needle. The needle was inserted through the transverse carpal ligament in line with the radial border of the fourth digit slightly ulnar to the palmaris longus tendon at the level of the distal wrist crease. Forty minutes was allowed for the median nerve block to reach complete effectiveness [23] and was verified using the Semmes-Weinstein monofilament test. The average monofilament score was 2.85 across the five digits before nerve block. About 40 minutes after nerve injection, little sensory impairment occurred in the ulnar distribution (score = 3.22), while the sensory score in the median distribution was greater than 6.15. The effects of nerve block lasted more than 6 hours with all subjects regaining normal hand function within 12 hours.

### Testing apparatus

**Figure 1:**
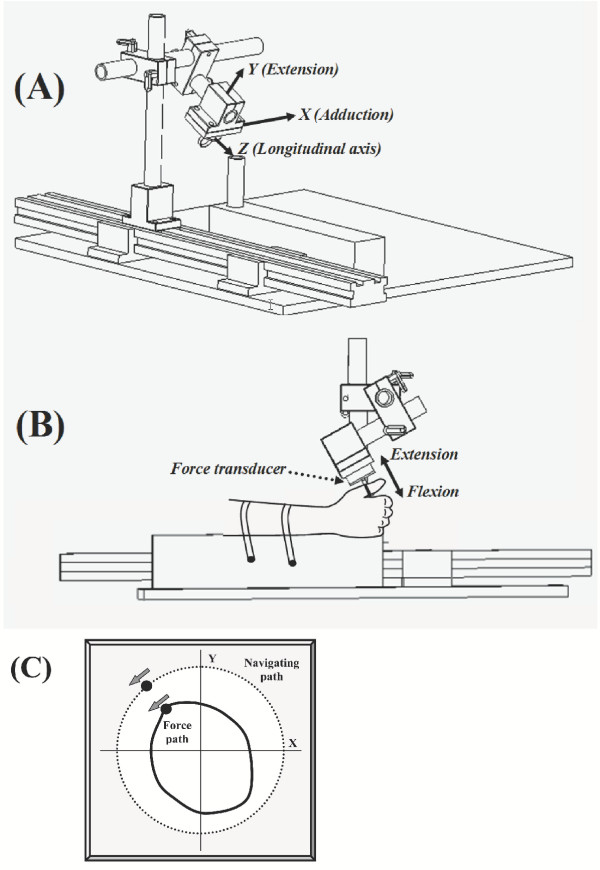
Schematic of experimental setup to measure thumb force production in the transverse plane. (A) 3D view. (B) Side view with hand and thumb in place. Thumb extension and flexion occur in parallel with the palm, and abduction and adduction occur in a plane perpendicular to the palm with abduction moves away from the palm. (C) Visual guide for circumferential force production.

The experimental apparatus was designed and constructed to measure maximum voluntary contraction forces of any digit at any point along the digit. Force application was possible in any direction within the transverse plane of the longitudinal axis of the digit. The apparatus consisted of position control accessories, a force transducer, and a custom fitted aluminum ring attached to the transducer (Figure 1A,1B). The transducer (Mini40, ATI Industrial Automation, NC, USA), capable of measuring 6 degrees of freedom forces and moments, was attached to a mounting clamp via an aluminum adapter plate while the aluminum ring was secured to the tool side of the transducer using a custom adapter. The ring served as a connection anchor for the transducer and the digit. The force transducer and ring attachment were positioned in a desired orientation using an aluminum slide rail, tubing, and lockable mounting clamps (80/20 Inc., Columbia City, IN, USA). The slide rail was secured to an aluminum base plate. Foam padded wooden blocks with two locking straps secured the arm to the base plate.

The analog outputs from the transducer were digitized using a 16-bit analog-to-digital converter (PCI-6031, National Instruments, TX, USA). The X (abduction/adduction) and Y (flexion/extension) force components in the transverse plane were displayed on the screen while the subject performed a force production task. The resolutions of the force transducer in its axial (flexion/extension) and horizontal (abduction/adduction) directions were 0.16 N and 0.08 N, respectively. A personal computer equipped with LabVIEW (National Instrument, TX, USA) was used for force data acquisition, display, and processing.

### Experimental procedures

Each subject was tested before and after median nerve block. The nerve block procedures were performed immediately after the completion of the first testing session. Post-block testing started after the verification of complete median nerve block, approximately 40 minutes after the injection. During each test, the subject was seated in a chair adjacent to the testing station modified with a wooden board to align their back vertically throughout the trials. The subjects rested their forearm on padded wooden blocks positioning their shoulder in approximately 60° of frontal plane abduction. Nylon straps fitted with plastic snap locking mechanisms secured the forearm and minimized the intervention of the elbow and shoulder during thumb force application. Subjects grasped a vertical dowel secured to the distal end of the wooden blocks in a midprone position. Formable thermoplastic braces were used to fix the elbow in 90° of flexion, and the wrist in 20° of extension and 0° of ulnar deviation. A metallic brace was used to fix the interphalangeal joint of the thumb in full extension. The aluminum ring was placed around the middle of the proximal phalanx and oriented to accommodate comfortable thumb position with the metacarpophalangeal joint flexed approximately 15°. Prior to testing, a line was drawn on the proximal phalange at the midpoint between the interphalangeal and metacarpophalangeal joints. The alignment of the ring with the circumferential line standardized the location of force application within and between subjects. As force application was at the middle of the proximal phalanx, mechanical action pertains to both the metacarpophalangeal and carpometacarpal joints. We chose the terminology of flexion/extension and adduction/adduction based on the mechanical action with respect to the metacarpophalangeal joint. With the thumb in the ring (Figure 1B), extension and flexion occurred in parallel with the palm, and abduction and adduction occurred in a plane perpendicular to the palm.

Each subject performed 15 circumferential MVC trials with randomized starting directions (Figure 1C). The subject was allotted 15 seconds to complete each circumferential trial, and was instructed to use the entire time allotted to traverse the perimeter of the ring once. A dot generated on the computer screen was programmed to traverse a circle within 15 seconds to provide the subject with directional feedback of their force application. Subjects were given 60 seconds of rest between each circumferential trial. Each subject was familiarized with the task with a few practice trials. Data were collected from each subject at 100 samples per second producing a total of 22,500 pairs of force components from the 15 circumferential trials. Our previous study [19] indicated that the testing protocol did not cause noticeable fatigue.

### Force envelope and quadrants

Data from multiple circumferential trials were accumulated to construct a force envelope. The procedures to generate a force envelope were as follows:

Cartesian force coordinates (*X*_*i*_, *Y*_*i*_) were transformed into polar coordinates (*R*_α_, α), where *R*_α _was the force magnitude at an angular position α. Each α was rounded to the nearest integer ranging from 0 to 359 degrees.

The maximum, *F*_α_, was determined from a string of *N *data points along each radial line defined by α. At the completion of the 15 trials, there were, on average, *N *= 63 data points on each radial line of α based on the distribution off the 22,500 data points around 360°.

A moving average with an interval of 10° was applied to the maximal series data *F*_α _(α = 0, 1, 2,..., 359) to obtain filtered maximal forces. These forces formed a *force envelope*.

**Figure 2:**
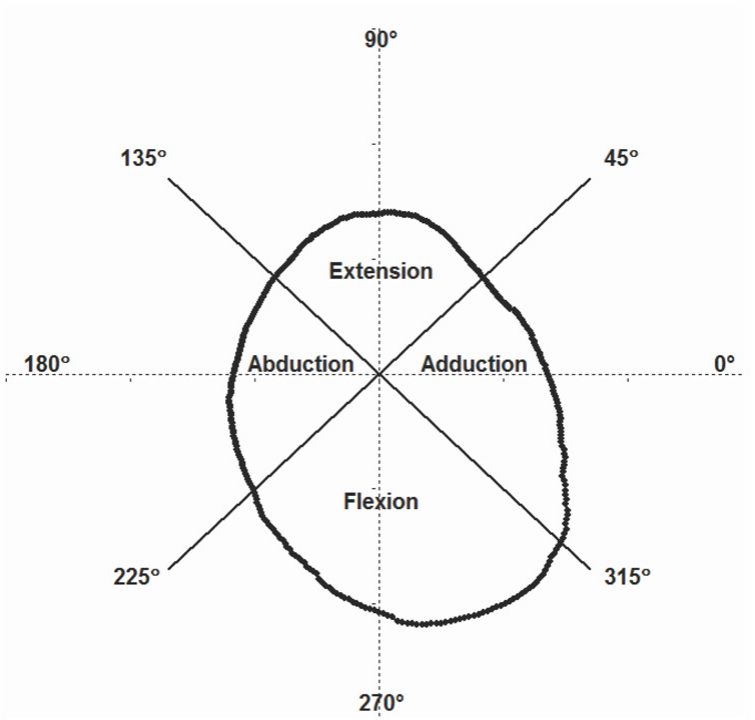
Division of force envelope into extension, abduction, flexion, and adduction quadrants.

The area formed by a force envelope was divided into adduction-extension, extension-abduction, abduction-flexion, and flexion-adduction quadrants by radial lines oriented at 0°, 90°, 180°, and 270° A *quadrant force *was represented using the mean magnitude of the forces in that quadrant. The areas of the entire envelope and each quadrant were calculated by summing the areas of individual arc sections formed by the polar coordinates of the force envelope. (Figure 2).

### Statistical Analyses

One- and two-factor repeated measures analyses of variance (ANOVA) were used to analyze outcome measures. The independent variables were testing SESSION (n = 2, i.e., pre- and post-block), force DIRECTION (n = 16), and force QUADRANT (n = 4), with SESSION as a repeated variable. Dependent variables were directional force, individual quadrant area and force envelope area. Statistical analyses were performed using SPSS 11 (SPSS Inc., Illinois) with statistical significance set at α = 0.05.

## Results

### Force envelope and directional forces

**Figure 3:**
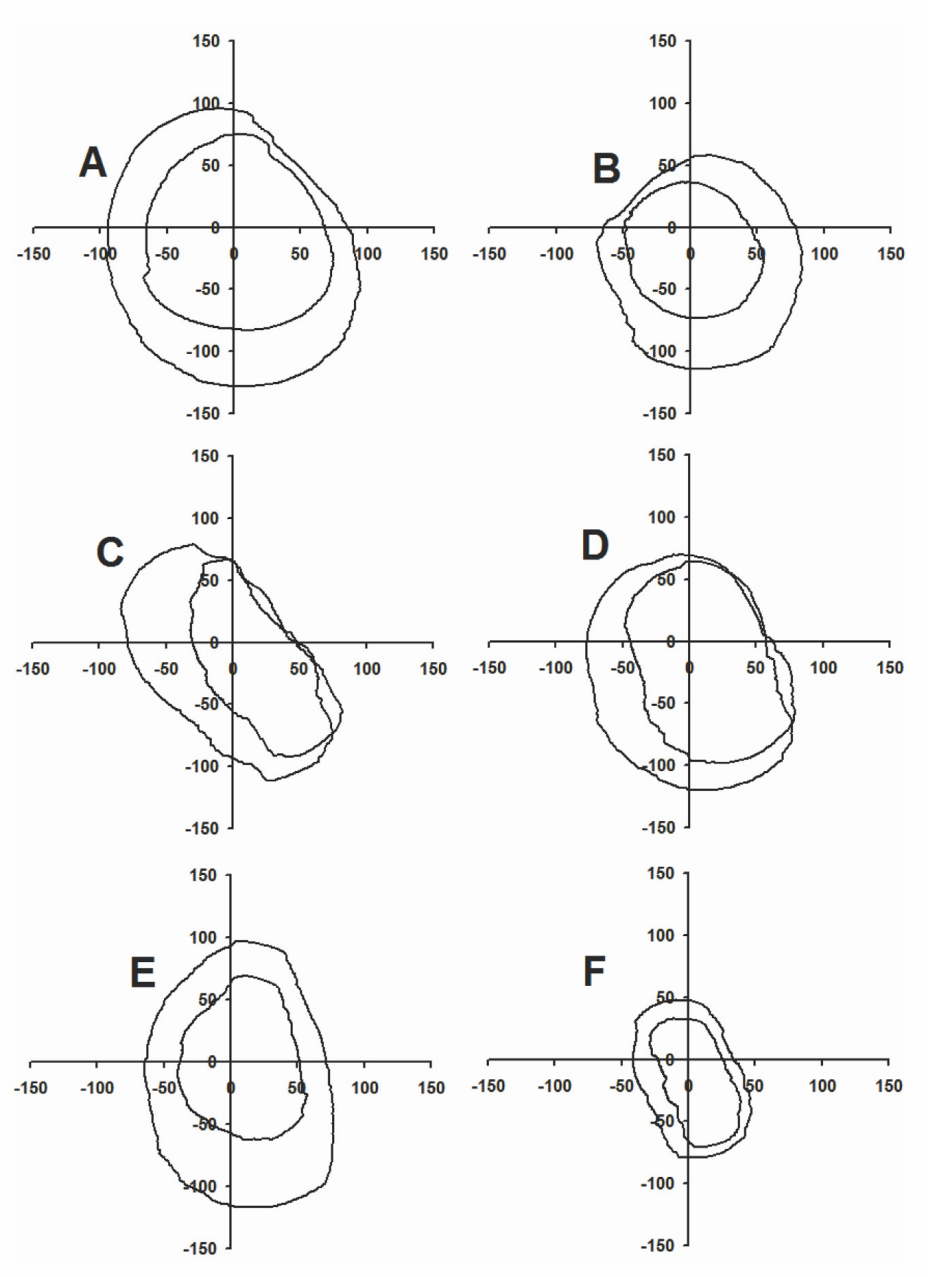
Force envelopes before and after median nerve block of subjects A, B, C, D, E, and F. For each subject, the inner envelope represents post-block results.

**Figure 4:**
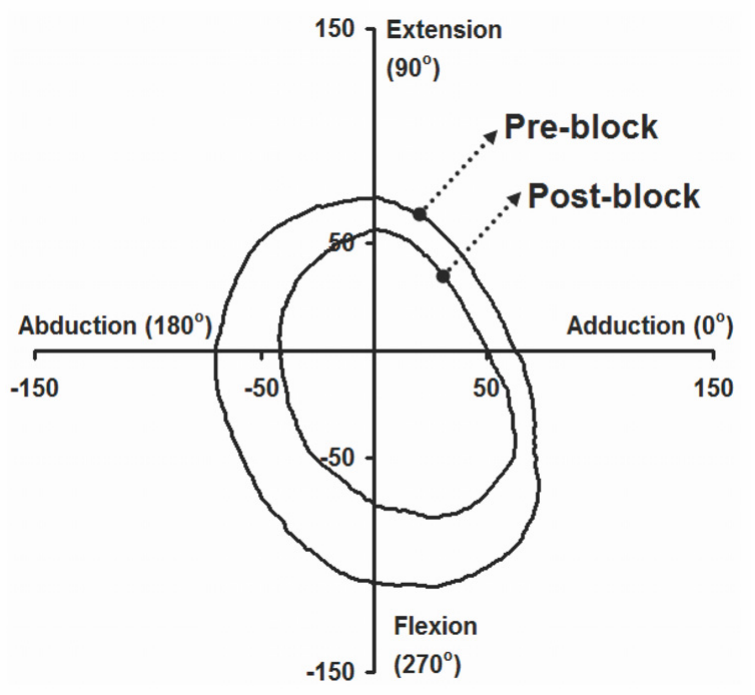
Average force envelopes produced by the thumb before and after median nerve block.

Figure 3 shows the force envelopes produced by each subject (A to F) before and after median nerve block. The post-block force envelope was inside the pre-block envelope for each subject, indicating a decrease in force magnitude in all directions after nerve block. Figure 4 shows the average pre- and post-block force envelope across all subjects. Force magnitudes were significantly reduced after nerve block (p < 0.001) resulting in significantly smaller force envelopes. The average decrease across all directions was 27.9%. A maximum decrease of 42.4% occurred at 199°, corresponding to a combined direction of abduction and slight flexion, while a minimum decrease of 10.5% occurred at 328° corresponding to a combined direction of adduction and slight flexion. Relative decreases at 0° (adduction), 90° (extension), 180° (abduction), and 270° (flexion) directions were 17.3%, 21.2%, 41.2% and 33.5%, respectively.

**Figure 5:**
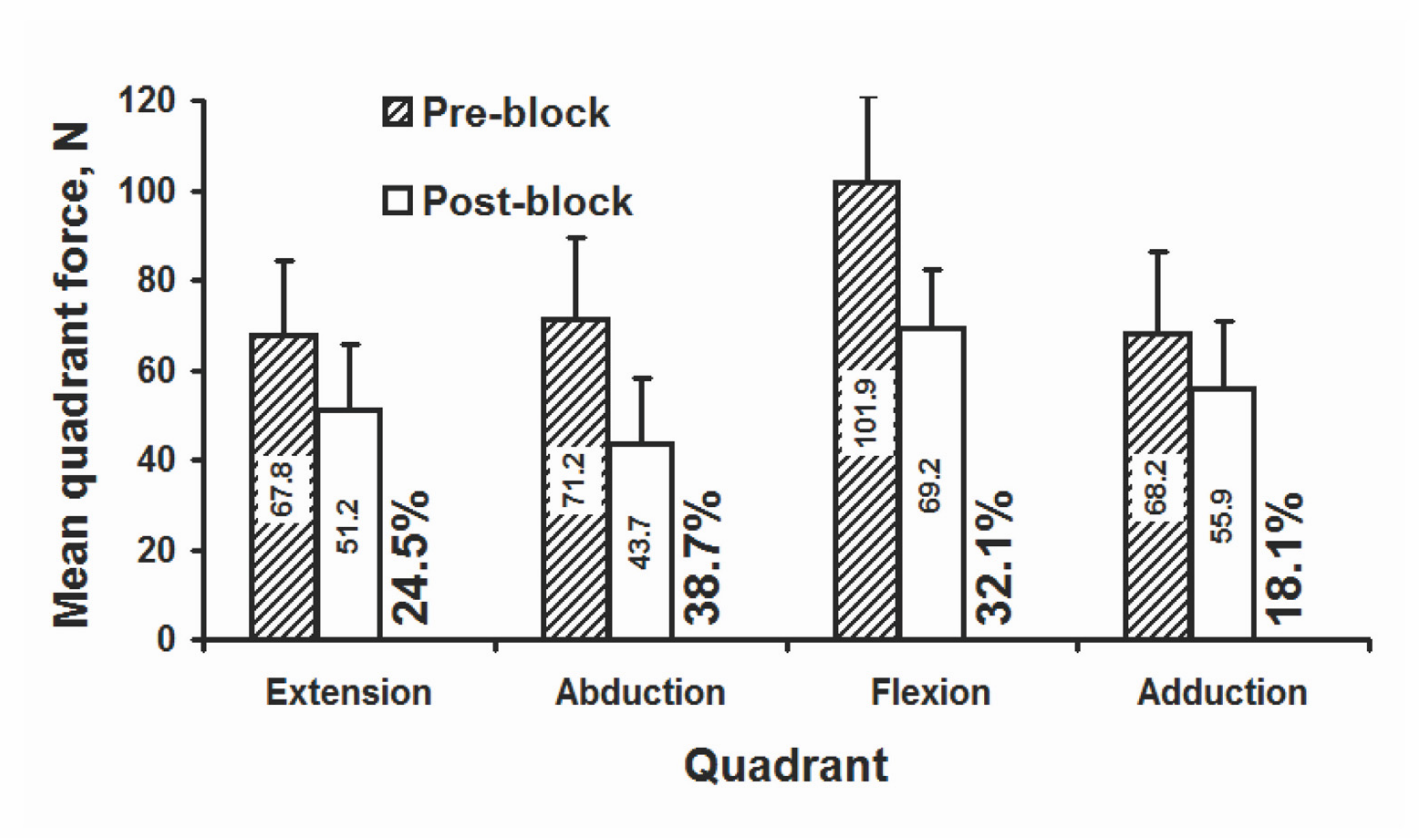
Average force magnitude, N, in individual quadrants. The percentage values denote the percent decreases of post-block forces relative to pre-block forces.

A single force in each quadrant was represented using the mean magnitude of the forces in that quadrant (see description in the Methods). The average quadrant forces were significantly decreased after nerve block (p < 0.001; Figure 5). The amount of decrease was also different between quadrants (p < 0.005). Relative decreases in mean quadrant forces were 24.5%, 38.7%, 32.1%, and 18.1% for extension, abduction, flexion, and adduction, respectively. The maximal decreases in mean quadrant force, 38.7%, occurred in the abduction quadrant.

### Force envelope areas and quadrant areas

**Figure 6:**
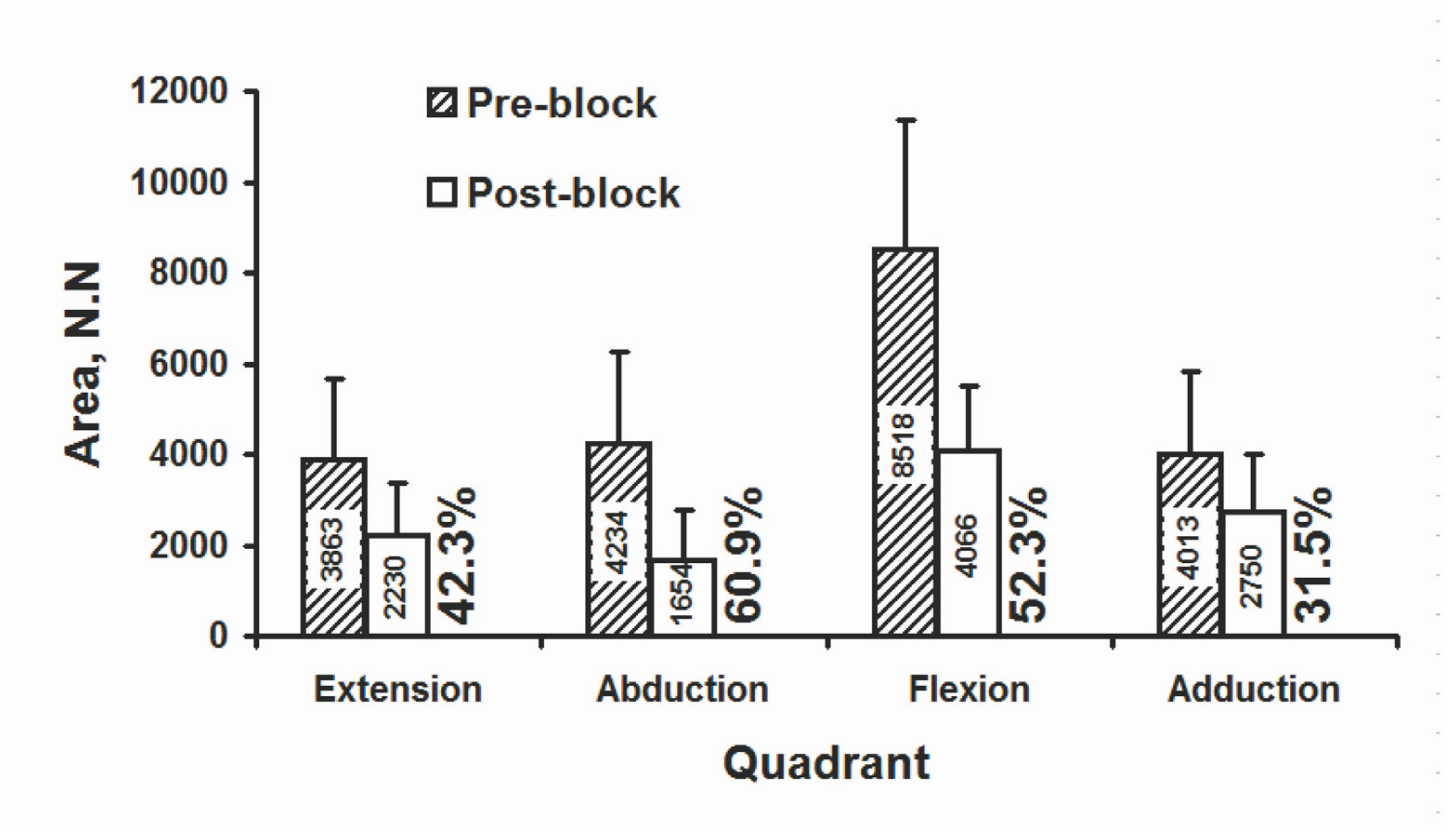
Area (N-N) of individual force quadrants, and percentage decrease after nerve block. The percentage values denote the percent decrease of post-block quadrant areas.

Areas enclosed by the post-block envelopes were significantly smaller than the pre-block envelopes (p < 0.001; Figure 4). Post-block force envelope area, 10700 ± 4474 N.N, was 51.9% of pre-block force envelope area, 20628 ± 7747 N.N. Quadrant area decreased significantly (p < 0.001; Figure 6). The maximal percentage decrease in area after nerve block was 60.9% in the abduction quadrant, followed by a 52.3% area decrease in the flexion quadrant.

## Discussion

In this study we simulated a lower median nerve lesion and evaluated the resultant thumb motor function deficit. Our internal control via pre- and post-block design offered a particular advantage of investigating the mechanical role of muscles innervated by a targeted nerve. The testing and analytical methods employed have provided advanced quantification of thumb motor function. The results have confirmed our initial hypotheses that greatest force decreases occurred in directions related to abduction, and that the post-block thumb force envelope area was smaller than the pre-block force envelope area.

Preferential force attenuation in the quadrants of abduction and flexion after median nerve block are in agreement with anatomical and neuromuscular features of the thumb. The median nerve innervates the abductor pollicis brevis, the opponens pollicis and superficial head of the flexor pollicis brevis, all of which contribute to the abduction and flexion of the thumb [4]; therefore, denervation of these muscles after median nerve block would cause the greatest force deficit related to median nerve function [5]. Additionally, as force application moved towards adduction, the force deficit decreased as neuromuscular control shifted from the median nerve to the ulnar nerve via the first dorsal interosseous and adductor pollicis brevis. Force deficit in extension was also comparably small as extension forces are mainly produced by the extensors pollicis brevis and longus originating in the forearm.

Our reported force decreases following a median nerve block (40.9% in abduction, 34.1% in flexion) were smaller than those reported in the literature. Kozin et al. [21] reported a 60% decrease in pinch strength after a median nerve block using mepivicaine hydrochloride [21]. Boatright and Kiebzak [20] reported an approximate 70% decrease in thumb abduction strength after median nerve block using Lidocaine [20]. Kaufman et al. [5] stated that a median nerve block with Lidocaine almost completely diminished force production in the abduction direction [5]. The discrepancy may be due to the anesthetic used and strength testing method. Although the sensory block appeared to be complete for each method, the motor capabilities of the muscles associated with the median nerve might or might not be completely eliminated. Such a result is largely dependent on a particular anesthetic, its concentration and dosage, as well as the efficacy of the injection technique at immersing the nerve. The methods of strength testing may also help explain the different magnitudes of strength deficit after the nerve block. All previous results were based on forces obtained in discrete direction(s), and focused exertions, while the current study utilized a method of force production in a continuous, circumferential and dynamic manner. Furthermore, thumb motor performance can be maintained despite the absence of certain individual muscles. For example, Britto and Elliot reported that the loss of abductor pollicis longus and extensor pollicis brevis in their two patients did not show functional compromise of strength and grip strength [24]. In a broader sense, the neuromuscular system has remarkable capabilities to accomplish the same motor function goal using different effectors and different goals using the same effectors, a phenomenon so called "motor equivalence" [25].

An unexpected finding from this study was that the force deficit occurred in all directions (Figure 4). In other words, the median nerve block caused reduced force production by those muscles not associated with the median nerve. Several potential explanations exist to describe such a phenomenon. First, the injection into the carpal tunnel at the wrist, although localized, potentially diffused into the intrinsic fascia of the hand partially compromising function of the ulnar nerve, which innervates the adductor pollicis. Although Semmes-Weinstein monofilament testing confirmed the continued sensation of the digits within the ulnar nerve distribution, it is not inconceivable that the injection could have contaminated the ulnar innervated muscles, the first dorsal interosseous and deep head of the flexor pollicis brevis [20]. Secondly, thumb force in any direction is produced by synergistic activation of the many intrinsic muscles, and as a result, the muscular deficiency associated with one direction may hinder the force production in other directions by other muscles [5,22]. For example, Kaufman et al. demonstrated that thumb muscles not innervated by the median nerve displayed lower electromyographical activation and shifted the direction of maximum activation after a median nerve block [5]. Labosky and Waggy showed that a radial nerve block caused a 53% decrease in thumb abduction strength because of the lack of stabilization of the radial innervated extensor muscles [22]. Consequently, deficiency of median innervated muscles inherently limits force production in other directions as neuromuscular switching is necessary to produce force in changing directions.

The median innervated muscles are the dominant abductors of the thumb metacarpophalangeal and carpometacarpal joint. The more than 50% residual abduction force found in this study suggests that the injection did not totally block the motor function of these muscles, even though a complete sensory loss was verified. This concurs with clinical observations of median compression neuropathy. Individuals with carpal tunnel syndrome complain of sensory dysfunction early in the disease process (at the beginning), while motor signs of thenar wasting and thumb weakness occur as the disease advances. The concept that the motor deficit is more resistant to peripheral median neuropathy than sensory loss has been well documented [23,26,27]. Butterworth et al. studied the temporal effects on sensory and motor blockade after injection of bupivacaine or mepivacaine, and found that sensory loss was complete but about a 20% compound motor action potential remained after 40 minutes [23].

In conclusion, we have incorporated a method for assessing thumb motor deficit based on strength measurement with a standard local anesthetic to investigate the effects of a simulated median neuropathy on thumb motor function. Median nerve block results in force deficits in all directions, with the most severe impairment in abduction and flexion. Future endeavors using this methodology can potentially further elucidate underlying pathomechanisms of peripheral neuropathies in all digits of the hand.
